# Insights Into Minimally Invasive Aesthetic Procedure-Defining Demographic Factors and Impact of the Treatment on Self-Perception: A Propensity-Matched Cross-Sectional Study

**DOI:** 10.1093/asjof/ojae111

**Published:** 2024-11-12

**Authors:** Mahmut Ozturk, Filipa Almeida Oliveira, Marta Ribeiro Teixeira, Sascha Wellenbrock, Baksan Tav, Maximillian Kueckelhaus, Philipp Wiebringhaus, Tobias Hirsch, Marie-Luise Aitzetmüller-Klietz, Matthias Aitzetmüller-Klietz

## Abstract

**Background:**

The lion's share of the research on facial aesthetic procedures focuses on visual results and patient satisfaction.

**Objectives:**

To characterize treated and untreated potential patients and the identification of determinants that influence the decision making as well as the impact of such treatments on patients’ self-perception.

**Methods:**

A cross-sectional, propensity-score-matched study on minimally invasive aesthetic procedures was performed through a survey comprising elements regarding demographics, conduct toward minimally invasive facial plastic interventions, as well as psychological well-being and social self-esteem domains of the validated Face-Q module.

**Results:**

Complete data were collected from 598 participants. Among those, 88% (*n* = 529) were females and 12% (*n* = 69) were males. Forty-six percent (*n* = 277) of the participants were below the age of 30, and 39% (*n* = 233) were between 30 and 45 years of age. Of the 598 participants, 22% (*n* = 134) of the participants had undergone a minimally invasive aesthetic procedure of botulinum toxin and/or hyaluronic acid injection, and 78% (*n* = 464) of the participants had never received any aesthetic treatment. Female gender, 30 to 45 years of age, self-employment, and an income over 75,000€ were significant predictors of undergoing minimally invasive aesthetic procedures. Among treated participants, the overall FACE-Q score as well as the scores for the social esteem and the psychological well-being domains improved significantly with the aesthetic treatment.

**Conclusions:**

This study highlights the major role of healthcare providers in better informing patients and the positive impact of facial aesthetic treatments on patients’ appearance as well as on psychological well-being and self-esteem.

**Level of Evidence: 5 (Risk):**

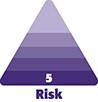

Reversing effects of gravity and signs of aging have been a major challenge through the ages, yet it has never been so popular. Over the last few decades, we have witnessed an extraordinary surge in minimally invasive aesthetic procedures such as botulinum toxin or hyaluronic acid injections performed.^1^ Although acknowledging the key role of technological advancements in making these procedures safer and more cost-effective, hence more accessible, it is also crucial to recognize the transforming societal perception of beauty and its impact on individuals’ self-impression.

Although even just the decision phase of undergoing a minimally invasive aesthetic procedure is complex enough and encompasses an innumerable number of individual, societal, and economical factors, the lion’s share of the research on this subject focuses on results and patient satisfaction. Yet the characterization of treated and untreated potential patients and the identification of determinants that influence the decision making as well as the impact of such treatments on patients’ self-perception remain unelucidated.

Comprehension of the incentives that motivate people to undergo or avoid minimally invasive aesthetic procedures by the healthcare providers as well as the industry could improve individualized and efficacious measures toward patient care and satisfaction as well as potentially identify patients with high risk of dissatisfaction.^2^ Hence, we sought to characterize treated and untreated potential patients and the identification of determinants that influence the decision making as well as the impact of such treatments on patients’ self-perception.

## METHODS

A cross-sectional study on minimally invasive aesthetic procedures was performed between March and September 2022 through an online questionnaire that comprised elements regarding demographics, conduct toward minimally invasive facial plastic interventions as well as psychological well-being and social self-esteem domains of the validated FACE-Q module.^3^ Participant recruitment was performed through social media. Gender, age, history of depression, relationship status, working status, level of education, the scale of the city lived in, and level of income were used in propensity score matching (PSM) as baseline demographic and patient/participant characteristics to achieve a balanced comparison of the treated and nontreated patient groups.

Excluding the incomplete assessments, data from 598 participants who answered all 40 questions could be collected completely. Among these 598, 134 participants constituted the treatment group and 464 participants belonged to the nontreated group.

Interpretations regarding the

1. preoperative FACE-Q scores of both the treated and nontreated groups,
2. preoperative and postoperative FACE-Q scores of treated patients,
3. reasons to avoid minimally invasive aesthetic treatments,
4. role of the demographic characteristics and motivations in undergoing the treatment,
5. role of financial stand in undergoing aesthetic treatments, and
6. potential effect of such treatments on psychological well-being were sought as the main objectives of the study.

IRB approval was waived by Westphalia–Lippe Physicians’ Chamber.

### Statistical Analysis

Continuous data are described using medians and interquartile ranges (IQRs), and categorical data are presented as frequencies and percentages. PSM was implemented to achieve a balanced comparison of the treated and nontreated groups in order to control for confounding and baseline imbalances regarding baseline demographics and participant characteristics. The evaluation of balance for each variable between the 2 treatment groups was performed using the standardized mean difference (SMD). SMD values <0.2 were considered as indicating good balance between the propensity-matched treatment groups on a given variable. PSM was performed 1 to 3, where each treated participant was matched to 3 nontreated participants. Optimal nearest-neighbor caliper matching without replacement was implemented. The caliper width was set to 0.2 times the standard deviation of the logit of the propensity scores. The comparison of the overall sum score and domains between the propensity-matched cohorts was performed using median regression with a robust standard error to account for clustering of participants within propensity-matched sets. The results from median regression are presented as adjusted differences in medians with corresponding 95% CI and *P*-value.

The comparison of scores and domains before and after treatment within the treated participants was performed using the nonparametric Wilcoxon signed-rank test for paired data. Multivariable logistic regression modeling was implemented to identify significant independent predictors of treatment (the propensity score model), with results presented as adjusted odds ratios, 95% CIs, and *P*-values. The comparison of demographics and participant characteristics by money spent on treatment was performed using Fisher's exact test.

All statistical analyses were performed using Stata (version 17, StataCorp LLC, College Station, TX). A 2-tailed *P* < .05 was implemented for determining statistical significance.

## RESULTS

### Characteristics

Complete data could be collected from 598 participants. Among those, 88% (*n* = 529) were females and 12% (*n* = 69) were males. Forty-six percent (*n* = 277) of the participants were below the age of 30, and 39% (*n* = 233) were between 30 and 45 years of age. A history of depression was present in 15% (*n* = 87) of participants. Of all participants, 19% (*n* = 114) were single, 47% (*n* = 280) were in a relationship, and 33% (*n* = 195) were married. Seventy-four percent (*n* = 444) of the participants were working as employees, and 5% (*n* = 29) were self-employed. Forty-one percent (*n* = 247) of the participants had an educational level up to a bachelor, 14% (*n* = 86) had a bachelor, and 44% (*n* = 265) had a master’s degree or higher. Forty-eight percent (*n* = 288) of the participants lived in a city with >100,000 residents. Thirty-two percent (*n* = 190) earned between 25,000 and 50,000€ (Table 1). Of the 598 participants, 22% (*n* = 134) of the participants had undergone a minimally invasive aesthetic procedure of botulinum toxin and/or hyaluronic acid injection and 78% (*n* = 464) of the participants had never received any aesthetic treatment.

**Table 1.** Comparison of Propensity-Matched Groups

Variable	Before matching			After matching (1-3)		
	Treatment (*n* = 134) *n* (%)	Nontreated (*n* = 464) *n* (%)	SMD	Treatment (*n* = 111) *n* (%)	Nontreated (*n* = 333) *n* (%)	SMD
Gender						
Male	7 (5.2)	62 (13.4)	0.28	7 (6.3)	33 (9.9)	0.13
Female	127 (94.8)	402 (86.6)		104 (93.7)	300 (90.1)	
Age, years						
<30	44 (32.8)	233 (50.2)	0.42	43 (38.7)	146 (43.8)	0.14
30-45	71 (53.5)	162 (34.9)		54 (48.7)	144 (43.2)	
45-60	17 (12.7)	51 (11)		13 (11.7)	37 (11.1)	
>60	2 (1.5)	18 (3.9)		1 (0.9)	6 (1.8)	
History of depression	26 (19.4)	61 (13.2)	0.17	20 (18)	49 (14.7)	0.09
Relationship status						
Single	27 (20.2)	87 (18.8)	0.09	24 (21.6)	63 (18.9)	0.15
In a relationship	60 (44.8)	220 (47.4)		50 (45.1)	154 (46.3)	
Married	44 (32.8)	151 (32.5)		37 (33.3)	113 (33.9)	
Other	3 (2.2)	6 (1.3)		0 (0)	3 (0.9)	
Employment status						
Student	16 (11.9)	94 (20.3)	0.35	16 (14.4)	63 (18.9)	0.14
Employed	103 (76.9)	341 (73.5)		88 (79.3)	253 (76)	
Self-employed	12 (9)	17 (3.7)		6 (5.4)	13 (3.9)	
Jobless	0 (0)	6 (1.3)		0 (0)	0 (0)	
Retired	3 (2.2)	6 (1.3)		1 (0.9)	4 (1.2)	
Educational attainment						
No training/vocational/technician's or equivalent	49 (36.6)	198 (42.7)	0.21	43 (38.7)	133 (39.9)	0.1
Bachelor's degree	15 (11.2)	71 (15.3)		14 (12.6)	52 (15.6)	
Master’s degree or higher	70 (52.2)	195 (42)		54 (48.7)	148 (44.4)	
Location of residence						
Large city (>100,000)	65 (48.5)	223 (48.1)	0.1	55 (49.6)	167 (50.2)	0.02
Medium city (20,000-100,000)	35 (26.1)	106 (22.8)		27 (24.3)	78 (23.4)	
Small town or village (<20,000)	34 (25.4)	135 (29.1)		29 (26.1)	88 (26.4)	
Yearly gross income						
<25,000€	26 (19.4)	107 (23.1)	0.43	23 (20.7)	77 (23.1)	0.19
25,000-50,000€	30 (22.4)	160 (34.5)		28 (25.2)	99 (29.7)	
50,000-75,000€	34 (25.4)	122 (26.3)		31 (27.9)	91 (27.3)	
75,000-100,000€	22 (16.4)	43 (9.3)		14 (12.6)	38 (11.4)	
>100,000€	22 (16.4)	32 (6.9)		15 (13.5)	28 (8.4)	

After PSM, the overall sum score is significantly higher in the treatment group (median = 58, IQR: 48-63) when compared with the nontreated group (median = 55, IQR: 46-62; adjusted difference = 3; 95% CI: 0.19-5.81; *P* = .036). There was no significant difference in the social esteem domain or the mental domain between matched treated and nontreated patients. SMD, standardized mean difference.

### Predictors of Undergoing a Treatment

After adjusting for all variables in the multivariable model, female gender was associated with significantly higher odds of treatment compared with male gender among all participants (adjusted odds ratio = 3.46; 95% CI: 1.44-8.33; *P* = .006). Compared with other age groups, participants between 30 and 45 years of age were significantly more likely to undergo a treatment (adjusted odds ratio = 1.89; 95% CI: 1.14-3.14; *P* = .014). Similarly, self-employment was associated with higher odds of undergoing treatment compared with working as an employee (adjusted odds ratio = 2.77; 95% CI: 1.13-6.79; *P* = .025). Also, yearly income from 75,000 to 100,000€ (adjusted odds ratio = 3.34; 95% CI: 1.59-7.02; *P* = .001) and >100,000€ (adjusted odds ratio = 3.45; 95% CI: 1.51-7.87; *P* = .003) were significant predictors of the treatment, whereas history of depression, relationship status, employment status, educational attainment, and location of residence were not (Table 2).

**Table 2.** Multivariable Logistic Regression of Predictors of Treatment (Propensity Score Model)

Variable	Adjusted odds ratio	95% CI	*P*-value
Gender			
Male	Reference		
Female	3.46	1.44-8.33	.006^a^
Age, years			
<30	Reference		
30-45	1.89	1.14-3.14	.014^a^
45-60	1.25	0.56-2.81	.583
>60	Cannot calculate		
History of depression	1.6	0.92-2.78	.096
Relationship status			
Single	1.54	0.81-2.92	.191
In a relationship	1.4	0.83-2.36	.202
Married	Reference		
Other	3.6	0.66-19.7	.139
Employment status			
Student	0.52	0.21-1.24	.141
Employed	Reference		
Self-employed	2.77	1.13-6.79	.025^a^
Jobless	Cannot calculate		
Retired	Cannot calculate		
Educational attainment			
No training/vocational/technician's or equivalent	1.67	0.81-3.43	.164
Bachelor's degree	Reference		
Master’s degree or higher	1.55	0.78-3.08	.207
Location of residence			
Large city (>100,000)	1.25	0.74-2.12	.411
Medium city (20,000-100,000)	1.37	0.77-2.43	.282
Small town or village (<20,000)	Reference		
Yearly gross income			
<25,000€	2.03	1.03-4.03	.052
25,000-50,000€	Reference		
50,000-75,000€	1.5	0.82-2.72	.186
75,000-100,000€	3.34	1.59-7.02	.001^a^
>100,000€	3.45	1.51-7.87	.003^a^

Female gender, age between 30 and 45, self-employment status, and income over 75,000€ were identified as significant predictors of undergoing minimally invasive aesthetic procedures. ^a^Statistically significant.

### Impact on Psychological Well-Being

A history of depression was present in 15% (87/598) of the entire participants. Among the treatment group of 134 participants, 26 (19%) participants had either depression or history of depression. In 4 (15%) of these participants, symptoms of depression improved since the treatment, and according to 3 of these participants, this improvement was primarily due to the minimally invasive aesthetic treatment.

### Evaluation of the FACE-Q Scores in Matched Groups

After the PSM (1:3), a good balance was achieved between participants with all SMDs <0.2. It resulted in 111 participants in the treated group and 333 participants in the nontreated group. The participant characteristics and the SMDs are summarized in Table 1.

**Figure 1.**
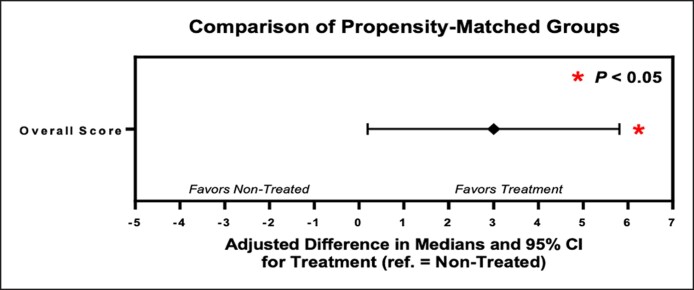
Comparison of propensity-matched groups. After PSM, the overall sum score is significantly higher in the treatment group (median = 58, interquartile range [IQR]: 48-63) when compared with the nontreated group (median = 55, IQR: 46-62; adjusted difference = 3; 95% CI: 0.19-5.81; *P* = .036). There was no significant difference in the social esteem domain or the mental domain between matched treated and nontreated patients.

The overall FACE-Q score prior to treatment was significantly higher in the treated group (median = 58, IQR: 48-63) when compared with the nontreated group (median = 55, IQR: 46-62; adjusted difference = 3; 95% CI: 0.19-5.81; *P* = .036). However, this significant difference was not observed when each domain was analyzed separately (Figure 1, Table 3).

**Table 3.** Comparison of FACE-Q Scores Among Propensity-Matched Groups

Outcome	Treatment (*n* = 111)	Nontreated (*n* = 333)	Adjusted difference	95% CI	*P*-value
Overall score sum	58 (48-63)	55 (46-62)	3	0.19, 5.81	.036^a^
Social esteem domain	24 (21-28)	23 (19-27)	1	−0.6, 2.6	.221
Mental domain	31 (27-37)	32 (27-36)	−1	−2.9, 0.9	.314

Data are presented as median (interquartile range). Median regression was implemented with a robust standard error to account for clustering within propensity-matched sets to calculate the adjusted coefficient, 95% CI, and *P*-value. ^a^Statistically significant.

Among treated participants, the overall FACE-Q score improved significantly with the aesthetic treatment (before treatment median = 58, IQR: 48-63, after treatment median = 61, IQR: 52-67; *P* < .001). Similarly, among treated participants, the scores for the social esteem as well as the psychological well-being domains improved significantly from before treatment (median = 25, IQR: 21-28 and median = 31, IQR: 27-37) to after treatment (median = 26, IQR: 22-30 and median = 35, IQR: 30-39, respectively; *P* < .001; Table 4).

**Table 4.** Comparison of the Overall Score Before vs After Treatment in the Treatment Group (*n* = 134)

Outcome	Before treatment (*n* = 134)	After treatment (*n* = 134)	*P*-value
Overall score sum	58 (48-63)	61 (52-67)	<.001^a^
Social esteem domain	25 (21-28)	26 (22-30)	<.001^a^
Mental domain	31 (27-37)	35 (30-39)	<.001^a^

Data are presented as median (interquartile range). The Wilcoxon signed-rank test was implemented to compare paired data before vs after treatment. Among treated patients, the overall score changed significantly from before treatment (median = 58, IQR: 48-63) to after treatment (median = 61, IQR: 52-67; *P* < .001). Among treated patients, the social esteem domain score changed significantly from before treatment (median = 25, IQR: 21-28) to after treatment (median = 26, IQR: 22-30; *P* < .001). Among treated patients, the mental domain score changed significantly from before treatment (median = 31, IQR: 27-37) to after treatment (median = 35, IQR: 30-39; *P* < .001). ^a^Statistically significant.

### Comparison Demographics by Money Spent on Treatment in the Last 12 Months

Of the 134 participants who received an aesthetic treatment anytime in the past, 74 (55%) spent within the last 12 months <350€, 41 (31%) between 350 and 800€, 17 (13%) between 800 to 1500€, and 2 between 1500 and 2500€. Eight-five percent (*n* = 6) of the male participants spent <350€, whereas around 46% of the female participants spent >350€ in aesthetic procedures in the same period (*P* = .509). Although the amount of money spent on minimally invasive aesthetic treatments was similarly distributed among student and employed participants with <350€ constituting the majority, the percentage of self-employed participants who spent 350 to 800€ was higher than of those who spent <350€ (33.3% vs 50%; *P* = .36). There were no statistically significant associations between demographic variables, such as gender, age, history of depression, relationship status, employment status, level of education, location of residence, and yearly income and amount of money spent on treatment in the past 12 months (Table 5).

**Table 5.** Comparison Demographics by Money Spent on Treatment in the Last 12 Months

Variable	<350€ (*n* = 74)	350-800€ (*n* = 41)	800-1500€ (*n* = 17)	1500-2500€ (*n* = 2)	*P*-value
Gender					
Male	6 (85.7)	1 (14.3)	0 (0)	0 (0)	.509
Female	68 (53.5)	40 (31.5)	17 (13.4)	2 (1.6)	
Age, years					
<30	29 (65.9)	11 (25)	4 (9.1)	0 (0)	.789
30-45	35 (49.3)	23 (32.4)	11 (15.5)	2 (2.8)	
45-60	9 (52.9)	6 (35.3)	2 (11.8)	0 (0)	
>60	1 (50)	1 (50)	0 (0)	0 (0)	
History of depression					
Yes	15 (57.7)	9 (34.6)	2 (7.7)	0 (0)	.811
No	59 (54.6)	32 (39.6)	15 (13.9)	2 (1.9)	
Relationship status					
Single	14 (51.9)	10 (37)	2 (7.4)	1 (3.7)	.869
In a relationship	34 (56.7)	18 (30)	8 (13.3)	0 (0)	
Married	24 (54.6)	12 (27.3)	7 (15.9)	1 (2.3)	
Other	2 (66.7)	1 (33.3)	0 (0)	0 (0)	
Employment status					
Student	9 (56.3)	5 (31.3)	2 (12.5)	0 (0)	.36
Employed	60 (58.3)	28 (27.2)	14 (13.6)	1 (1)	
Self-employed	4 (33.3)	6 (50)	1 (8.3)	1 (8.3)	
Jobless	—	—	—	—	
Retired	1 (33.3)	2 (66.7)	0 (0)	0 (0)	
Educational attainment					
No training/vocational/technician’s or equivalent	31 (63.3)	13 (26.5)	4 (8.2)	1 (2)	.124
Bachelor's degree	4 (26.7)	9 (60)	2 (13.3)	0 (0)	
Master's degree or higher	39 (55.7)	19 (27.1)	11 (15.7)	1 (1.4)	
Location of residence					
Large city (>100,000)	37 (56.9)	18 (27.7)	9 (13.9)	1 (1.5)	.951
Medium city (20,000-100,000)	18 (51.4)	13 (37.1)	4 (11.4)	0 (0)	
Small town or village (<20,000)	19 (55.9)	10 (29.4)	4 (11.8)	1 (2.9)	
Yearly gross income					
<25,000€	13 (50)	11 (42.3)	2 (7.7)	0 (0)	.349
25,000-50,000€	20 (66.7)	8 (26.7)	2 (6.7)	0 (0)	
50,000-75,000€	18 (52.9)	9 (26.5)	5 (14.7)	2 (5.9)	
75,000-100,000€	12 (54.6)	4 (18.2)	6 (17.3)	0 (0)	
>100,000€	11 (50)	9 (40.9)	2 (9.1)	0 (0)	

Data are presented as *n* (%). *P*-values were calculated using Fisher's exact test.

### Evaluation of the Deterring Factors

A total of 193 participants (193/598, 32%) reported that they would not undergo a minimally invasive aesthetic treatment. The most common reasons for avoiding such treatments were not believing that such a treatment would change patient's life in any way (109/193, 56.5%), fear of looking “fake” (97/193, 50.3%), and the deterring examples in the media (such as complications; 53/193, 27.5%). These were followed by fundamentally being against aesthetic treatments (39/193, 20.2%), other reasons (10/193, 17.1%), and using alternative therapies (such as creams; 26/193, 13.5%). Except for “partner's negative opinion on such treatments” being significantly the least common cause (*n* = 3, *P* = .01), none of the aforementioned factors were significant causes of avoiding a treatment.

For a similar question in a separate section of the questionnaire, 45% of the participants answered with concerns related to the cost of treatment, and 30% reported that they had not found a suitable provider yet.

## DISCUSSION

The current study investigates the motivations and deterrents as well as the impact of undergoing a minimally invasive aesthetic procedure, with a particular focus on the utilization of proper statistical methods in a large group of participants. The findings elucidate different aspects regarding participant demographics, psychological well-being, self-perception, treatment-related changes, and discouraging variables.

Specific demographic factors were identified to have a significant correlation with the likelihood of undergoing facial aesthetic interventions. In alignment with the existing literature, female gender, self-employment status, and higher income levels were significantly associated with seeking treatment,^4^ demonstrating the influence of socioeconomic factors on aesthetic preferences. In contrast to the older cohorts, the age group where the signs of aging begin, notably the cohort between 30 and 45 years old, had a higher propensity of seeking aesthetic treatments, emphasizing the influence of the appearance of the aging manifestations rather than the age itself.

In this study, 2 separate domains of the established FACE-Q questionnaire, psychosocial well-being and self-esteem, were used, and the scores for each domain were both analyzed individually and together as an overall score. The evaluation of these scores demonstrated significantly higher overall FACE-Q scores for patients opting for the treatment compared with those who did not, highlighting a potentially stronger self-perception prior to treatment. Based on this finding, it could be speculated that the widely accepted theory that lower self-esteem or psychological well-being are motivators for seeking aesthetic treatment might not be accurate. However, this was only valid for the overall score, as a significant difference was not observed between treated and nontreated groups by means of their scores in each domain. The subgroup analysis of the treated participants also showed a significant improvement of overall FACE-Q as well as psychological well-being and self-esteem scores, revealing the positive impact of such treatments on self-perception.

The identification of elements discouraging patients from undergoing such treatments is pivotal in the field of aesthetic surgery. Despite the observed benefits among treated individuals in this study, one-third of participants were against these and similar treatments, with nearly half of those opposing believing that such interventions would yield no positive impact on their lives or would result in an unnatural appearance. This bears considerable significance for patient education and healthcare providers from an economic standpoint. It becomes crucial to convey to potential patients that although positive outcomes showcased in media may not always represent the norm, instances displaying unfavorable results are exceedingly rare. This underscores the necessity of conveying this understanding to prospective patients through various means.

Another important finding of the study was that two-thirds of the participants who never underwent an aesthetic treatment considered doing so. The main deterring factors were for 45% of the participants the cost of the treatment, and for 30% not yet having found a suitable provider. Primarily from the patient's standpoint, the inability to afford the treatment deemed necessary poses a risk of leading them toward locations and unreliable aesthetic providers. These economically appealing aesthetic providers/aesthetic chains may lack quality and an appropriate level of education, which may lead to more complications and unfavorable results in the media, also leading to an increase in the number of fearsome patients that avoid aesthetic procedures. Simultaneously, from the healthcare provider's standpoint, suboptimal pricing might result in missing out on a large number of potential patients they could otherwise reach. This double-edged challenge emphasizes the necessity for a balanced approach in pricing strategies to ensure accessibility without compromising quality and reliability in aesthetic treatments.^5^

Parallel to the existing literature,^6-8^ our study showed a positive impact of aesthetic treatments on psychological well-being, self-esteem. The subgroup analysis of the FACE-Q scores showed a statistically significant improvement of overall FACE-Q as well as psychological well-being and self-esteem scores. These results show that aesthetic treatments not only improve the overall appearance of patients but also positively impact their psychological well-being and self-esteem. Two participants even reported an improvement in depression due to aesthetic treatment. Considering the limitations inherent in the current study's methodology, confirmation of the reported results through prospective randomized trials or longitudinal follow-up studies is warranted.

### Limitations

Among the limitations of this study, the reliance on self-reported data collected from participants with online media access introduces the potential for both recall and selection biases. Furthermore, the single-country focus and a predominant representation from a specific ethnicity may limit the diverse representation within the study cohort. Additionally, although the questionnaire used in this study underwent validation for reliability and validity, its specific nature may not be appropriate for robust psychiatric inferences.

## CONCLUSIONS

Female gender, 30 to 45 years of age, self-employment, and an income over 75,000€ were significant predictors of undergoing minimally invasive aesthetic procedures, and among the treated participants, the overall FACE-Q score as well as the scores for the social esteem and the psychological well-being domains improved significantly with the aesthetic treatment. With these findings, the current study highlights the major role of healthcare providers in better informing patients, addressing individual concerns as well as inquiries comprehensively, and aligning patient expectations within realistic bounds and defines the significant demographic variables of seeking minimally invasive aesthetic procedures as well as its positive impact not only on patients’ appearance as well as on psychological well-being and self-esteem.
